# Complete Genome Sequence of the Feline Calicivirus 2280 Strain from the American Tissue Culture Collection

**DOI:** 10.1128/genomeA.00349-13

**Published:** 2013-06-20

**Authors:** Tomoichiro Oka, Hirotaka Takagi, Linda J. Saif, Qiuhong Wang

**Affiliations:** Food Animal Health Research Program, Ohio Agricultural Research and Development Center, Department of Veterinary Preventive Medicine, College of Veterinary Medicine, The Ohio State University, Wooster, Ohio, USAa; Department of Virology II, National Institute of Infectious Diseases, Tokyo, Japanb; Division of Biosafety Control and Research, National Institute of Infectious Diseases, Tokyo, Japanc

## Abstract

Feline calicivirus (FCV) is a highly contagious pathogen of cats that can be grown in cultured cells. FCV is used as a model to study nonculturable caliciviruses, such as noroviruses. We determined the complete genome sequence of the FCV 2280 strain from the American Tissue Culture Collection.

## GENOME ANNOUNCEMENT

Feline calicivirus (FCV) belongs to the genus *Vesivirus*, in the family *Caliciviridae*. It is a highly infectious pathogen of cats. The FCV genome contains an approximately 7.7-kb positive-sense single-stranded RNA with three open reading frames (ORFs). ORF1 encodes nonstructural proteins, and ORF2 and ORF3 encode structural proteins, the precursor VP1 and VP2, respectively (1). There are complete genome sequences of 14 FCV strains in GenBank (accession no. L40021, M86379, D31836, U13992, AF109465, AF479590, AY560113 to AY560118, DQ424892, and GU214989). Strain FCV 2280 was isolated in 1983 from a blood sample of a cat with Limping syndrome and is available from American Tissue Culture Collection (ATCC VR-2057) (1, 2). Currently, only the ORF2 gene of this strain is available in GenBank (accession no. X99445) (2). We analyzed the full-length genome of FCV 2280.

Crandell-Reese feline kidney cells were inoculated with FCV 2280 at multiplicity of infection (MOI) of 0.0001 and cultured in Eagle’s minimal essential medium supplemented with 2% heat-inactivated fetal bovine serum and antibiotics (100 units/ml penicillin and 100 µg/ml streptomycin). The virus was harvested at 56 h postinoculation for RNA extraction. cDNA was synthesized using a tagged oligo(dT) primer, TX30SXN (5′-GACTAGTTCTAGATCGCGAGCGGCCGCCCT_30_-3′) (3). The 5.4-kb 5′-end and 3-kb 3′-end cDNA fragments covering the complete genome were amplified with PrimeStar HS DNA polymerase (Takara) and were sequenced directly by primer walking using a set of FCV 2280-specific primers. The 5′-terminal sequence was obtained by using the 5′ rapid amplification of cDNA ends (5′-RACE) kit (Invitrogen).

The genome of FCV 2280 consists of 7,683 nucleotides (nt), excluding the poly(A) tail. It is predicted to encode three ORFs: ORF1 (nt 20 to 5311), ORF2 (nt 5314 to 7320), and ORF3 (nt 7317 to 7637). The 5′- and 3′-untranslated regions were 19 nt and 46 nt long, respectively. Compared to the ORF2 sequence in GenBank (accession no. X99445), our sequence showed 20 nt differences, resulting in 11 amino acid substitutions in the predicted precursor VP1 protein. Differences between current and previous studies under virus propagation conditions and analysis may have precipitated these changes: (i) infection dose, MOI 0.0001 versus 10, (ii) cultivation period, 56 h versus ~12 h, (iii) PCR enzymes, PrimeStar HS DNA polymerase versus AmpliTaq DNA polymerase (PerkinElmer), and (iv) sequencing procedure of PCR amplicon, direct sequencing versus cloning (2).

A protein BLAST search with the predicted ORF1-encoded protein revealed the highest identity (95%) with FCV strain UTCVM-NH1 (accession no. AY560113), detected in 1993. These two strains shared 88% and 98% amino acid identity in the predicted precursor VP1 and VP2 proteins, respectively. The 5′-end 45 nt (nt 1 to 45) and the ORF1-ORF2 junction region 33 nt (nt 5307 to 5339 of FCV 2280) were completely conserved (100% identity), and nt 2420 to 2448 (in FCV 2280) were highly conserved (28 out of 29 nt) among the 15 complete FCV genomes available, suggesting a critical role of these motifs in the FCV life cycle. These conserved sequences might be targeted for the design of FCV universal primers for diagnostic purposes.

### Nucleotide sequence accession number.

The genome sequence of the FCV 2280 strain has been deposited in GenBank under the accession no. KC835209.
